# Rosai-Dorfman Disease Originating from Nasal Septal Mucosa

**DOI:** 10.1155/2015/232898

**Published:** 2015-05-10

**Authors:** Abdulvahap Akyigit, Hadice Akyol, Oner Sakallioglu, Cahit Polat, Erol Keles, Ozkan Alatas

**Affiliations:** ^1^Elazig Training and Research Hospital, Department of ENT, 23100 Elazig, Turkey; ^2^Elazig Training and Research Hospital, Department of Pathology, 23100 Elazig, Turkey; ^3^Firat University Hospital, Clinic of ENT, 23100 Elazig, Turkey; ^4^Elazig Training and Research Hospital, Department of Radiology, 23100 Elazig, Turkey

## Abstract

Rosai-Dorfman disease is a rarely seen disease with unknown etiology. Extranodal involvement is most commonly seen in the head and neck region. Histopathologically, it is characterized by histiocytic cell proliferation. This paper presents a case of a 15-year-old male patient who presented with nasal obstruction and was surgically treated for a mass filling in the left nasal meatus that was diagnosed to be Rosai-Dorfman disease by histopathological examination.

## 1. Introduction

Rosai-Dorfman disease (RDD), which was first described by Rosai and Dorfman in 1969, is a nonneoplastic lymphoproliferative disease with unknown etiology and pathogenesis [1].

Painless bilateral cervical lymphadenopathy is present in most of the patients and is generally accompanied by fever, leukocytosis, increased sedimentation rate, and hypergammaglobulinemia [1]. Extranodal involvement is identified in 43% of the patients, in addition to lymphadenopathy [2]. The disease has a variable clinical spectrum changing from spontaneous remission to fatal vital organ involvement [3].

Definitive diagnosis can be performed by histopathological examination, which reveals a significant proliferation of sinus histiocytes and the observation of lymphocytes and erythrocytes phagocytized by histiocytes [4–6].

The current study presents the case of a 15-year-old male patient who had a mass originating from the septal mucosa and completely filling the left nasal meatus. The diagnosis of extranodal localized Rosai-Dorfman disease was made after the histopathological examination of the surgical specimen following surgical excision.

## 2. Case

**Figure 1:**
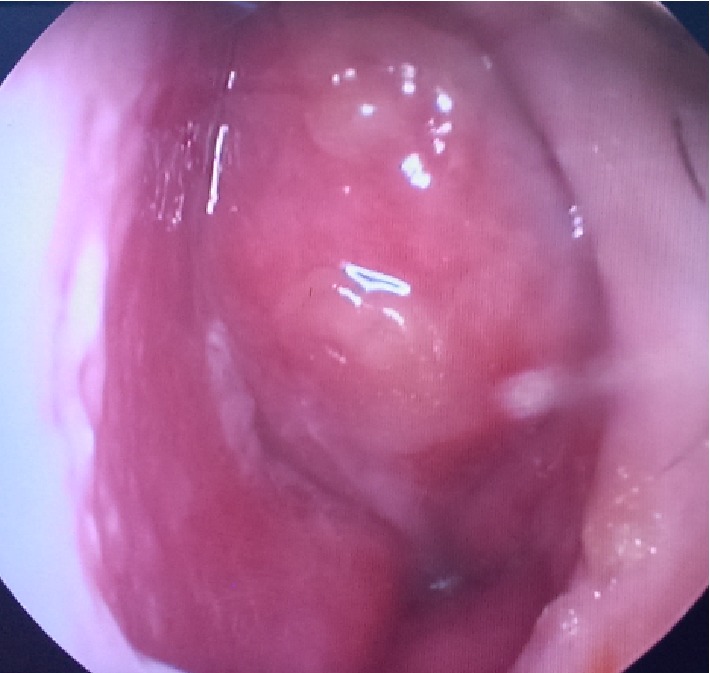
Endoscopic image of the mass in the left nasal meatus.

**Figure 2:**
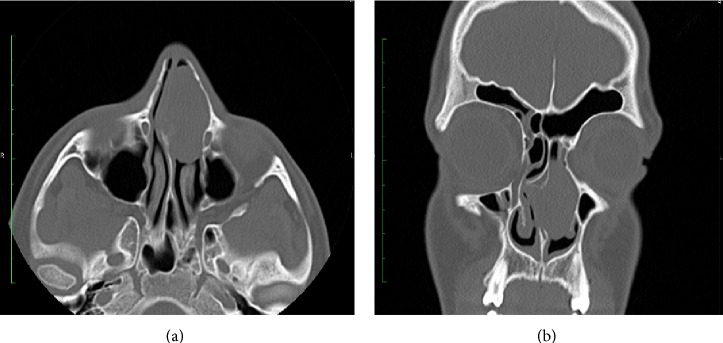
The image of the mass at the left nasal cavity in axial (a) and coronal (b) paranasal CT.

**Figure 3:**
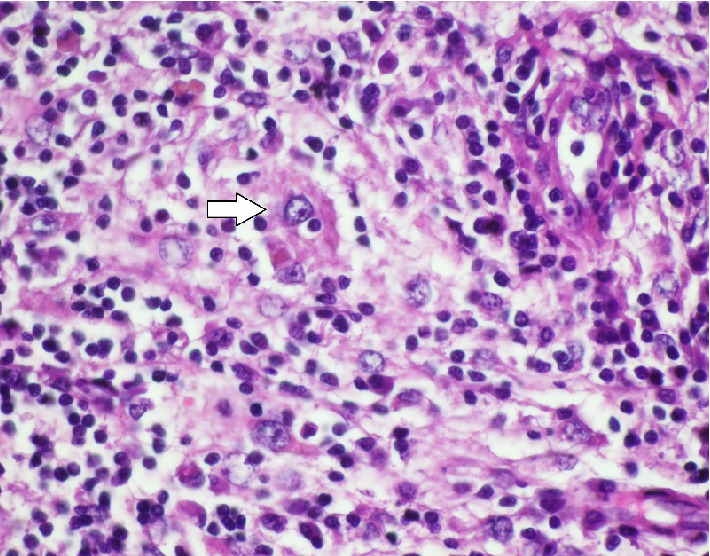
The image of the histiocyte that was observed to have emperipolesis in the histopathological examination (white arrow) (H&E, ×4).

A 15-year-old male patient presented to the outpatient clinic of our department due to difficulty in breathing from the nose. A mass filling in the left nasal meatus was detected via an anterior rhinoscopic examination. The endoscopic nasal examination revealed that the mass originated from the septum mucosa and it had no association with the base of the skull (Figure 1). A solid lesion measuring 37 × 23 mm, originating from the nasal septal mucosa, was observed in the computed tomography images of the paranasal sinuses, which obliterated the left meatus and created a deviation by shifting the nasal septum to the right (Figure 2). Therefore, a punch biopsy was obtained from the solid lesion at the left nasal meatus. Since the biopsy result was reported to be a benign lesion, the mass was endoscopically excised through the left nasal meatus under general anesthesia. The mass was observed to originate from the nasal septal mucosa and was associated with the skull base and medial concha. The mass was excised through the left nasal meatus. The histopathological examination revealed an intense histiocytic infiltration. The histiocytes had large and vesiculated nuclei and emperipolesis was observed (Figure 3). There was patchy fibrosis in the background. The histiocytes were S100(+), CD68(+) and negatively stained with CD1a. Histopathological and immunohistochemical findings were reported to support the diagnosis of Rosai-Dorfman disease with extranodal involvement.

The patient was well at the sixth month follow-up visit.

## 3. Discussion

Rosai-Dorfman disease (RDD) is a disease with unknown etiology, characterized by histiocytic proliferation of the lymphatic sinuses, which primarily involves the lymph nodes, but can also demonstrate extranodal involvement [7]. The head and neck regions are the nodal fields that are most frequently involved. Most commonly affected extranodal fields are the skin, nasal cavity, eyes, and bone structure [2]. The presence of nasal cavity involvement in the current case is found to be compatible with the findings in the literature. Although an occult chronic infection or an infectious agent or immune response against an antigen has been considered the cause of histiocyte proliferation in the pathogenesis of the disease, no causative association with any etiological agent could be demonstrated [2].

Rosai-Dorfman disease is histopathologically diagnosed. The lymphatic sinus is invaded by plasma cells and histiocytes, and the structure of the lymphatic sinus is partially or completely damaged as a result. The most significant histopathological property of the disease is the detection of phagocytized lymphocytes and at times other cells, such as plasma cells and erythrocytes, with undamaged structures in the cytoplasm of the histiocytes, which is also known as emperipolesis [8]. Emperipolesis is seen less frequently in extranodal disease. The histopathological examination of the specimen demonstrated that histiocytes had performed emperipolesis in this case.

Histiocytic proliferative diseases are among the differential diagnoses. Lymphoma, tuberculosis, sarcoidosis, and reactive sinus hyperplasia should be considered. In addition, due to the localization and the development of a nasal mass, primarily nasopharyngeal carcinoma and olfactory neuroblastoma should also be considered in the differential diagnosis. Juvenile xanthogranuloma, which is a non-Langerhans cell histiocytic proliferation, was excluded due to its localization, while Langerhans cell histiocytosis, which is on the other end of the spectrum, is differentiated from RDD with the absence of emperipolesis, the presence of a smaller and notched nucleus, and CD1a positivity. In this case, S100 and CD68 were strongly positive, while CD1a was negative. Nasopharyngeal carcinoma is differentiated from RDD with its cell type and aggressive behavior. Chronic inflammatory diseases such as tuberculosis and sarcoidosis were excluded when clinical, laboratory, and histopathological findings were considered together. Furthermore, a marked difference in the histopathology and simultaneous CD4 and CD8 positivity excluded lymphoma.

Since Rosai-Dorfman disease generally has a benign course and is self-limited, it does not require treatment most of the time [9]. However, it may have an aggressive course in a very few number of cases and might be fatal [2]. The ideal type of treatment regime could not be determined since the disease is very rare and spontaneous remission is possible. Among the wide-spectrum treatment options are steroids, radiation, chemotherapy, interferon, acyclovir, monoclonal antibody treatment, and thalidomide [9–11]. Surgical treatment is indicated when a life-threatening enlargement of the lymph nodes or situations that cause functional impairment occur [9]. In the present case, the mass completely filled the left nasal meatus and caused difficulty in breathing through both nasal meatus while deviating the septum to the right; thus it was excised surgically.

Extranodal RDD should also be considered in nasal cavity tumors, although it is very rarely seen. There is no established ideal protocol in the treatment of this disease; however, it would be appropriate to surgically excise the lesion when it causes a functional disorder.
